# Recombinant Humanized Collagen Enhances Secreted Protein Levels of Fibroblasts and Facilitates Rats’ Skin Basement Membrane Reinforcement

**DOI:** 10.3390/jfb16020047

**Published:** 2025-02-01

**Authors:** Shijia Ye, Boyu Chen, Lakshmi Jeevithan, Haoze Yang, Yaqi Kong, Xiaozhen Diao, Wenhui Wu

**Affiliations:** 1 Department of Marine Biopharmacology, College of Food Science and Technology, Shanghai Ocean University, Shanghai 201306, China; m220351098@st.shou.edu.cn (S.Y.); 2234116@st.shou.edu.cn (B.C.); jlakshmi@ucam.edu (L.J.); m230351143@st.shou.edu.cn (H.Y.); m230351113@st.shou.edu.cn (Y.K.); 2 Department of Biomaterials Engineering, Faculty of Health Sciences, UCAM-Universidad Catolica San-Antonio de Murcia, 30107 Murcia, Spain; 3 Putuo Branch of International Combined Research Center for Marine Biological Sciences, Zhoushan 316104, China; 4 Marine Biomedical Science and Technology Innovation Platform of Lin-Gang Special Area, Shanghai 201306, China

**Keywords:** recombinant humanized collagen, HFF-1, skin basement membrane, ultraviolet injury

## Abstract

Collagen and its peptides exhibit remarkable antioxidant activity, superior biocompatibility, and water solubility, making them a significant research focus in skin care. Hence, the recombinant humanized collagen types I, III, and XVII complexed with niacinamide were developed to address damage in human foreskin fibroblasts (HFF-1) caused by ultraviolet radiation and to evaluate basement membrane proteins in a rat skin model. The Cell Counting Kit-8 (CCK-8) assay showed that higher concentrations of the complex increased the survival of damaged cells by approximately 10% and 22%, respectively, compared to the normal group after 16 and 48 h of treatment. Further biochemical analyses using ELISA and immunofluorescence (IF) confirmed that the complex enhanced the expression of collagen type IV, laminin, P63, and transforming growth factor-β (TGF-β) in the damaged cells. Additionally, the complex boosted the activity of the basement membrane in rat skin and stimulated the secretion of integrin, laminin, and perlecan. Overall, the recombinant humanized collagen complex effectively reinforced the skin’s basement membrane.

## 1. Introduction

Collagen is the most abundant protein in the animal body, accounting for one-third of the total protein in the human body and three-fourths of the net weight of the skin. It is mainly secreted by fibroblasts, and it is the most common component of the extracellular matrix (ECM) [1]. It is a tightly packed triple helical structure formed by the entanglement of three identical or different alpha-chains, and the three polypeptide chains have a Gly-X-Y tripeptide sequence, where X and Y are usually proline and hydroxyproline residues [2]. According to Grandview Research, the global collagen market was valued at USD 17,258 million in 2022 and is expected to reach USD 22,622 million by 2027 with a compound annual growth rate of 5.42% [3]. Collagen is used in vivo not only largely to maintain the structural integrity of tissues and organs but also to interact with specific receptors mediating signaling [4,5]. Due to their excellent antioxidant activity, biocompatibility, water solubility, accessibility, and ease of digestion and absorption, collagen and its peptides play a crucial role in corneal treatment, wound healing, and skin anti-aging [6,7]. As a result, it has a broad potential for application in the pharmaceutical and cosmetic industries. The collagen market size in the healthcare segment is expected to reach USD 11,117 million by 2027 [3].

The skin is the second largest barrier in the body, following blood vessels, and serves as the external shield against environmental physical, chemical, and microbial factors [8]. The skin comprises two morphologically distinguishable compartments: the epidermis and dermis [9]. The dermis provides essential support for the epidermis; its primary cell type is the fibroblast. Fibroblasts produce ECM proteins (e.g., collagen, fibronectin, and elastin), which are the main substances of the dermis and are responsible for the skin’s elasticity and tensile strength [10]. The basement membrane (BM) is a complex network of ECM macromolecules that connect the epidermis and dermis, physically separating these two layers and mainly serving as a stabilizing agent [11]. The main components of the BM are structural scaffold matrix proteins such as collagen types IV, VII, XVII, heparan sulfate polysaccharide, laminin, and fibronectin [12,13]. Among them, laminin and fibronectin are found in the thylakoid layer of the BM [14]. Additionally, integrins, keratins, TGF-β, etc., have important functional roles in the BM [15,16].

Currently, collagen has become a new favorite in the cosmetics industry. The most common types of collagen on the market are I, II, and III, mainly used for topical and oral administration [17,18]. However, most collagens on the market are derived from animal sources, which poses a safety and pathogenic risk in cosmetics due to the complexity of tracing the source and the possibility of transmitting pathogen vectors [19,20,21]. Recombinant humanized collagen (RHC) is free of animal components, avoiding mutagenicity, immunogenicity, and risk of infection. Studies have shown that RHC has better repair and protective effects on wound healing and ultraviolet (UV)-induced skin photoaging compared to collagen from animal sources [22,23]. The possible reasons for the low market share of RHC are its complex production process, lack of clear consensus on the production system, and imperfect regulatory system [24].

However, few studies have been reported on the synergistic effect of different types of RHC. Therefore, in this study, different type I, III, and XVII recombinant humanized collagens with nicotinamide were prepared to investigate the impact of different complex concentrations on the expression of basement membrane (BM) proteins and related cytokines. We employed both an in vitro cellular model of UVC damage and a rat skin model to investigate the reinforcing effects of the RHC complex on the BM.

## 2. Materials and Methods

### 2.1. Chemicals and Reagents

Human foreskin fibroblasts (HFF-1) were offered by Zhejiang Meisen Cell Technology Co., Ltd. (CTCC-001-0346, Meisen CTCC, Jinhua, China). Dulbecco’s Modified Eagle Medium (DMEM) high glucose and penicillin–streptomycin (pen–strep) were purchased from Pricella (Wuhan, China). Fetal bovine serum (FBS) was purchased from Meisen CTCC (Jinhua, China). Trypsin-EDTA was purchased from Gibco (Grand Island, NY, USA). Phosphate-buffer solution (PBS) was obtained from Servicebio Technology (Wuhan, China). CCK-8 was obtained from AbMole BioScience (Hoston, TX, USA).

### 2.2. Cell Culture

HFF-1 cells were cultured in T25 flasks in a humidified incubator (Rundu Biotechnology, Shanghai, China, Herocell 180N) with 5% CO_2_ at 37 °C in DMEM high glucose containing 15% (*v*/*v*) fetal bovine serum and 1% (*v*/*v*) penicillin–streptomycin. The cells were passaged every 2–3 days and used between passages 7 and 11 for all experiments.

### 2.3. Recombinant Humanized Collagen Complexes Synthesis

Recombinant humanized type I collagen (rhCOLI, Mw: 110–120 kDa), recombinant humanized type III collagen (rhCOLIII, Mw: 10~43 kDa), recombinant humanized type XVII collagen (rhCOLXVII, Mw: 10~23.8 kDa), and nicotinamide were provided by Jiangsu Chuangjian Medical Technology Co., Ltd. (Changzhou, China). And the amino acid sequence of three proteins is shown in the Supplementary Table S1. Recombinant Humanized Collagen (RHC) complex was prepared by dissolving collagen and nicotinamide in deionized water in a mass ratio of rhCOLI:rhCOLIII:rhCOLXVII:Nicotinamide = 50:200:5:1.

### 2.4. UVC Injury

HFF-1 was seeded at 1 × 10^4^ cells/0.1 mL cell density with complete culture medium as described in Section 2.2 in 96-well plates. After incubation for 24 h, each well was replaced with 0.1 mL of PBS. Then, the cells were irradiated at 550 mm distance from the center of an ultraviolet-C (UVC) lamp (length 894 mm, power 30 W, wavelength 254 nm) set within a super clean bench (BioClean Bench, Shangguang, Shanghai, China, SW-CJ-2F) [25].

### 2.5. CCK-8 Assay

The HFF-1 cells were adjusted to 1 × 10^4^/mL with complete culture medium as described in Section 2.2 in 96-well plates for the cytotoxicity assay. After cell attachment, the culture medium was replaced with DMEM containing the RHC complex, and the cells were incubated for 48 h. Cell viability was determined by a CCK-8 kit to evaluate cytotoxicity.

Cells were treated as described in Section 2.4 and then the culture medium was replaced with DMEM containing the RHC complex (rhCOLI:rhCOLIII:rhCOLXVII:Nicotinamide = 50:200:5:1) in different concentrations (0.01, 0.05, 0.1, 0.5, and 2 mg/mL). The blank group was served without test samples. Both the blank and the rhCOLXVII group were UVC-irradiated and the normal group was not UVC-irradiated without adding other samples. After incubating the cells for 16 and 48 h, the cell viability was determined by a CCK-8 kit to assess cell proliferation.

### 2.6. Rats’ Skin Sample Preparation

Wistar rats were purchased from Shanghai SLAC Laboratory Animal Co., Ltd. (Shanghai, China). Animal research protocols and procedures for this study were approved by the Animal Care and Use Committee of Shanghai Ocean University (Permit No. SHOU-DW-2023-063). All methods followed the relevant guidelines and regulations of Scientific and Ethical Care and Use of Laboratory Animals at Shanghai Ocean University.

Rats of average size were chosen for the experiment, ensuring an equal distribution of both female and male subjects. The dorsal region of each rat was shaved using a depilatory razor to create two distinct areas, each with dimensions of 2 × 2 cm (4 cm^2^). After that, a hair removal cream was meticulously applied to delete any remaining hair. Following a 24-h recovery period, 42 rats were randomly divided into seven groups. The groups consisted of a blank group (0 mg/mL RHC complex, *n* = 6), a control group (0.5 mg/mL rhCOLXVII, *n* = 6), and five experimental groups (*n* = 6 each) that received treatments of 0.01, 0.05, 0.1, 0.5, and 2 mg/mL RHC complex, with the composition of rhCOLI:rhCOLIII:rhCOLXVII:Nicotinamide being 50:200:5:1. The specific experimental grouping is shown in Table 1. In each group, 120 μL of the corresponding drug to be tested was uniformly applied to the depilatory area on the back of the rats every day, and the day of administration was defined as the first day of the test, once a day for 14 days. The rats were euthanized on days 7 and 14, and the dorsal skin tissues were excised for histological analysis.

### 2.7. Hematoxylin-Eosin Staining (H&E) Observation of BM

Rat dorsal skin models were fixed in 10% (*v*/*v*) formalin at 4 °C overnight and dehydrated in a graded ethanol series for paraffin embedding. Sections 3 mm thick were cut and mounted on slides. Tissue sections were deparaffinized in xylene, rehydrated in a decreasing graded ethanol series, and then stained with hematoxylin and eosin (H&E). Stained slides were photographed using a microscope (Olympus, Tokyo, Japan) and BM morphology was visualized using Slide Viewer (3DHISTECH Ltd., Budapest, Hungary; Version 2.6.0) software.

### 2.8. ELISA

HFF-1 cells were cultured in T25 flasks until the density reached around 90%, inoculated in 96-well plates with 1 × 10^4^ cells per well, modeled, and administered according to the method described in Section 2.4. The specific experimental grouping is shown in Table 2. The cell culture media were pipetted after 16 and 48 h, centrifuged at 3000 rmp for 20 min, and the supernatants were used for extracellular protein assay. Cell supernatants were evaluated for type I collagen (COLI), type IV collagen (COLIV), fibronectin (FN), and laminin (LN) using respective ELISA kits (Meimian, Yancheng, China) and for transforming growth factor-β (TGF-β) and P63 using respective ELISA kits (Bioswamp, Wuhan, China) according to the manufacturer’s instructions.

Rat skin tissue specimens (0.2 g) were taken from each group and fully homogenized with 1.8 mL PBS in an ice bath using a handheld homogenizer, centrifuged at 10,000 rmp at 4 °C for 15 min, and the supernatant was taken to analyze the levels of integrin, LN, and perlecan proteins using ELISA kits (Maisha, Yancheng, China).

### 2.9. Immunofluorescence (IF)

HFF-1 cells were seeded in 24-well plates with 5 × 10^4^ cells per well and treated as described in Section 2.4. After treatment, cells were washed with PBS and fixed with 4% (*w*/*v*) paraformaldehyde (PFA). IF staining was performed with type I collagen (Proteintech, Wuhan, China, 14695-1-AP), type IV collagen (Huabio, Hangzhou, China, HA500197), fibronectin (Proteintech, Wuhan, China, 23498-1-AP), and laminin (Proteintech, Wuhan, China, 15613-1-AP). HFF-1 cells were incubated overnight at 4 °C with primary antibody and then washed with PBST. After rinsing, cells were incubated in the dark for 1 h at 37 °C with an Alexa Fluor^®^ 488 goat anti-rabbit IgG (Beyotime, Shanghai, China, A0423) to detect primary antibodies. To stain the nuclei, HFF-1cells were incubated for 5 min with DAPI (Beyotime, Shanghai, China). Moreover, the cells were analyzed using an inverted fluorescence microscope (Olympus, Tokyo, Japan). Fluorescence intensity was analyzed by ImageJ (NIH, Bethesda, USA; Version 1.46).

Skin tissue samples fixed with 4% (*w*/*v)* PFA were embedded in paraffin, sliced, and fixed on slides. The samples were blocked with 3% (*w*/*v*) bovine serum albumin (BSA) and PBS, and incubated overnight at 4 °C with laminin beta 1 antibody (Proteintech, Wuhan, China, 23498-1-AP), integrin alpha 5 antibody (Abcam, Cambridge, MA, USA, ab150361), and Heparan Sulfate Proteoglycan 2 antibody (Abcam, Cambridge, MA, USA, ab315029). After washing, the cells were incubated with Anti-rabbit lgG (H + L) secondary antibodies (CST, Danvers, MA, USA, #4412) for 50 min, and the nuclei were stained with DAPI (Boosun Biotechnology, Beijing, China). LN, integrin, and perlecan protein expression were observed by immunofluorescence microscopy (Olympus, Tokyo, Japan).

### 2.10. Statistical Analysis

Data were represented as mean ± SD. Statistical analysis was performed using one-way ANOVA or *t*-tests. Multiple comparisons of means were performed using Fisher’s least significant difference (LSD) and Duncan’s test. Statistical significance was set at *p* < 0.05. All statistical analyses were performed using SPSS 27.0. Mapping was conducted using GraphPad Prism (La Jolla, CA, USA; Version 9.5).

## 3. Results

### 3.1. The RHC Complex Alleviated the UVC Injury in HFF-1 Cell Viability

**Figure 1:**
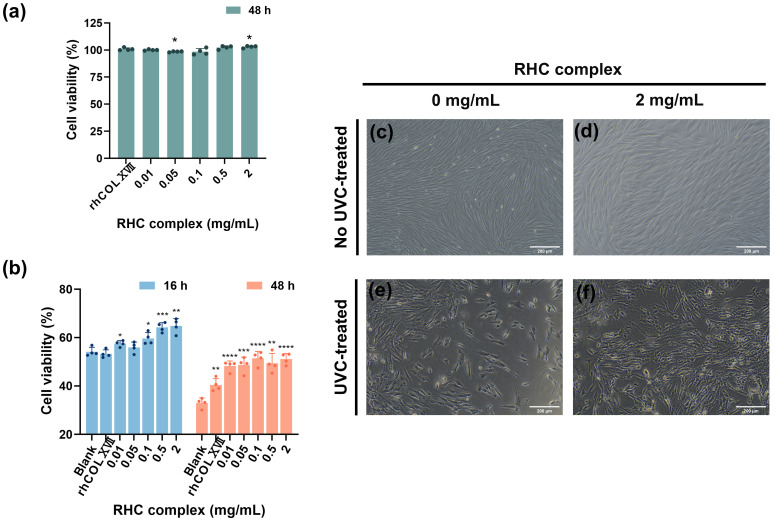
Cell viability alterations in HFF-1 cells under diverse experimental conditions. (**a**) Cell viability (HFF-1) as a function of RHC complex concentration. Data are the mean values from *n* = 4 replicates, expressed as a percentage of cell viability, normalized to the normal (no sample treated)= 100% cell viability, ±standard deviation. Statistical analysis of the variance of the mean values was assessed by *t*-test (* *p* < 0.05). (**b**) Cell viability (HFF-1) as a function of RHC complex concentration after irradiation (UVC = 0.3 J/cm^2^). Data are the mean values from *n* = 4 replicates, expressed as a percentage of cell viability, normalized to the normal (no UVC-treated) = 100% cell viability, ±standard deviation. Statistical analysis of the variance of the mean values was assessed by *t*-test (* *p* < 0.05; ** *p* < 0.01; *** *p* < 0.001; **** *p* < 0.0001). Cell morphology of HFF-1 treated with (**c**) 0 mg/mL RHC complex; (**d**) 2 mg/mL RHC complex for 48 h; (**e**) 0 mg/mL RHC complex plus irradiation (UVC = 0.3 J/cm^2^); and (**f**) 2 mg/mL RHC complex plus irradiation (UVC = 0.3 J/cm^2^) for 16 h. (Scale bar: 200 μm).

HFF-1 cells were cultured using the RHC complex (rhCOLI:rhCOLIII:rhCOLXVII:Nicotinamide = 50:200:5:1) to observe cell proliferation. Figure 1a shows that after normalization with the blank group (0 mg/mL), the cell proliferation rate of serum-free medium containing different concentrations of RHC complexes (0.01, 0.05, 0.1, 0.5, and 2 mg/mL) after 48 h of treatment was greater than 95%, indicating that the RHC complex had no cytotoxicity. Compared to the 0.5 mg/mL rhCOLXVII, treatment with 2 mg/mL significantly promoted the proliferation of HFF-1 cells. After 48 h of drug treatment (Figure 1c,d), HFF-1 cells had elongated fibers, tightly arranged morphology, abundant cytoplasm, and intact cell membranes. Interestingly, adding the drug did not alter the original cell morphology.

UVC irradiation significantly inhibited the proliferation of HFF-1 cells, resulting in noticeable cell detachment, which became more pronounced over time. The survival of UVC-exposed cells measured at 16 and 48 h dropped to approximately 55% and 35% of the normal group, respectively. Figure 1b shows that the incorporation of the RHC complex following UVC irradiation contributed to mitigating the suppression of HFF-1 cell proliferation, resulting in an approximate increase in cell viability of 10% and 22% after 16 and 48 h of treatment with 2 mg/mL samples, respectively, when compared to the blank group (0 mg/mL). At both 16 and 48 h, this improvement was more pronounced with higher concentrations of the complexes. However, treatment with rhCOLXVII for 16 h did not lead to an increase in the survival of damaged cells. Microscopically (Figure 1e,f), it can be seen that the shedding of HFF-1 cells was notably less than that of the blank group (0 mg/mL), and the cell morphology appeared more robust after 16 h of drug treatment.

### 3.2. The RHC Complex Inhibited the Down-Expression of BM Protein in UVC-Damaged HFF-1 Cells

As target BM proteins, COLI, COLIV, FN, and LN were chosen based on their epidermal origin. For this purpose, a series of RHC complexes (rhCOLI:rhCOLIII:rhCOLXVII:Nicotinamide = 50:200:5:1) was tested based on prior investigations.

**Figure 2:**
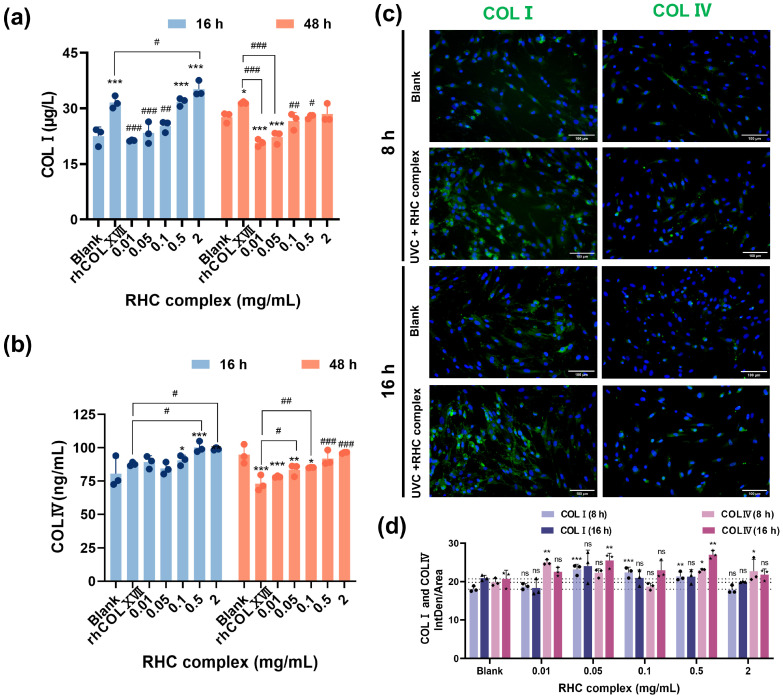
Expression of COLI and COLIV in UVC-damaged HFF-1 cells. (**a**) COLI level in UVC-damaged HFF-1 cell cultures treated with different concentrations of the RHC complex. (**b**) COLIV level in UVC-damaged HFF-1 cell cultures treated with different concentrations of the RHC complex. (**c**) IF micrographs of HFF-1 stained with COLI (green), COLIV (green), and DAPI (blue) after treatment with 2 mg/mL RHC complex plus irradiation (UVC = 0.3 J/cm^2^). (**d**) COL I and COLIV quantitative analysis from IF imaging after RHC complex treatment at different concentrations plus UVC irradiation (UVC = 0.3 J/cm2). Data represent mean of *n* = 3 replicates ± SD. (* *p* < 0.05, ** *p* < 0.01 and *** *p* < 0.001 compared with the blank group. # *p* < 0.05, ## *p* < 0.01, and ### *p* < 0.001 compared with the rhCOLXVII group. ns indicates no significant difference).

As shown in Figure 2a, the extracellular COLI level of cells damaged by UVC light (0.3 J/cm^2^) after 16 and 48 h of incubation in a culture medium containing RHC complexes showed an increasing trend with increasing concentration. Compared with the blank group (0 mg/mL), the higher concentration (0.5–2 mg/mL) of the sample was more effective than the control group in promoting the secretion of COLI after 16 h of treatment. To further analyze the mechanisms of the RHC complex in cells with UVC-induced damage, we conducted a quantitative IF analysis of COLI. As demonstrated in Figure 2c,d, at 8 h, the RHC complex of 0.05–0.5 mg/mL significantly increased COLI expression compared to the blank group, although this enhancement was absent at 16 h. The application of the RHC complex on UVC-damaged HFF-1 cells for 16 h resulted in an increase in COLIV secretion. Notably, the levels observed in the high concentration group (0.5–2 mg/mL) were significantly elevated compared to those in the control group. This promotion was positively correlated with the concentration of the samples at both 16 and 48 h. The mean intensity of IF (Figure 2c,d) also indicated that the expression of COLIV in the cells of the treated group was higher than that of the blank group. This finding further substantiated that 0.5–2 mg/mL of the RHC complex could enhance collagen synthesis in UVC-damaged skin fibroblasts, contributing to the reinforcement of the BM.

**Figure 3:**
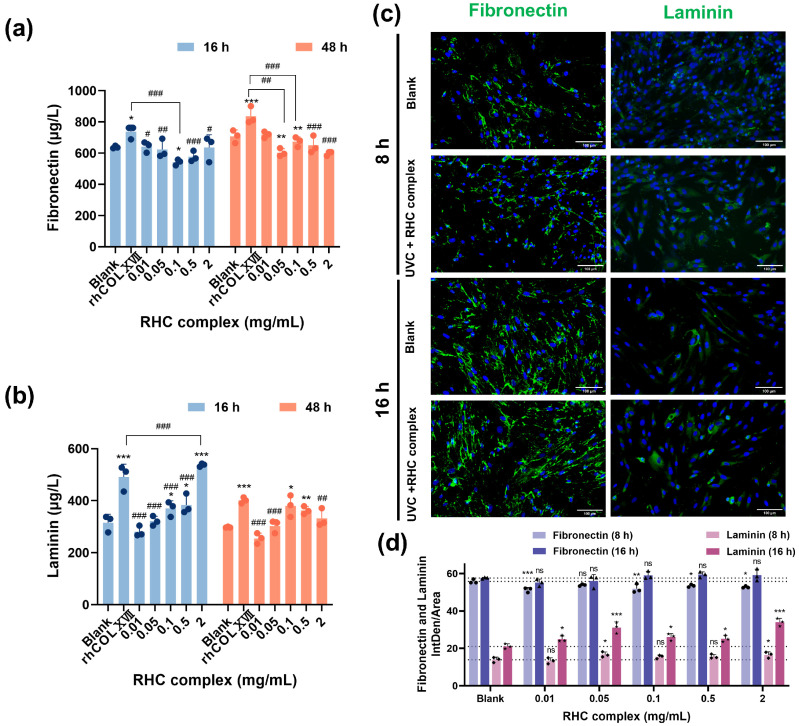
Expression of FN and LN in UVC-damaged HFF-1 cells. (**a**) FN level in UVC-damaged HFF-1 cell cultures treated with different concentrations of RHC complex. (**b**) LN level in UVC-damaged HFF-1 cell cultures treated with different concentrations of RHC complex. (**c**) IF micrographs of HFF-1 with FN (green), LN (green), and DAPI (blue) after treatment with 2 mg/mL RHC complex plus irradiation (UVC = 0.3 J/cm^2^) (Scale bar: 100 μm). (**d**) FN and LN quantitative analysis from IF imaging after RHC complex treatment at different concentrations plus UVC irradiation. All groups were irradiated (UVC = 0.3 J/cm^2^), the rhCOLXVII group used culture media containing 0.5 mg/mL rhCOLXVII, and the blank group was the group that used 0 mg/mL of culture medium. Data represent the mean of *n* = 3 replicates ± SD. (* *p* < 0.05, ** *p* < 0.01 and *** *p* < 0.001 compared with blank group; # *p* < 0.05, ## *p* < 0.01, ### *p* < 0.01 compared with rhCOLXVII group. ns indicates no significant difference).

To investigate whether FN and LN play a role in the reinforcement of damaged cells after the action of the RHC complex, we detected the expression of FN and LN in HFF-1 cells and performed IF analysis. As shown in Figure 3a, compared with the blank group, cells receiving UVC injury in culture media containing different concentrations of RHC complexes showed no significant increase in FN contents after 16 and 48 h, but there was a significant increase after rhCOLXVII treatment. The results of IF (Figure 3c,d) also illustrated that the average intensity of IF was not significantly enhanced in the treatment group compared to the blank group. The data presented in Figure 3c,d demonstrate a positive correlation between the levels of LN in the cell culture media and the concentration of the sample. Notably, the LN levels were significantly elevated in the higher concentration group (2 mg/mL) compared to the blank group (0 mg/mL) and were also greater than those observed in the rhCOLXVII group following the treatment of UVC-damaged HFF-1 cells with the RHC complex for 16 h; however, this difference diminished after 48 h of treatment. Furthermore, the mean IF intensity (Figure 3c,d) also showed that the LN contents in the damaged cells were significantly higher than that in the blank group after 16 h of the RHC complex treatment. This finding is significant as it corresponds with the expression results of COLIV, highlighting the role of specific binding interactions between cellular LN and collagens in facilitating the subsequent upregulation of collagens.

### 3.3. The RHC Complex Inhibited the Down-Expression of Cell Growth Regulators by UVC Injury in HFF-1 Cells

**Figure 4:**
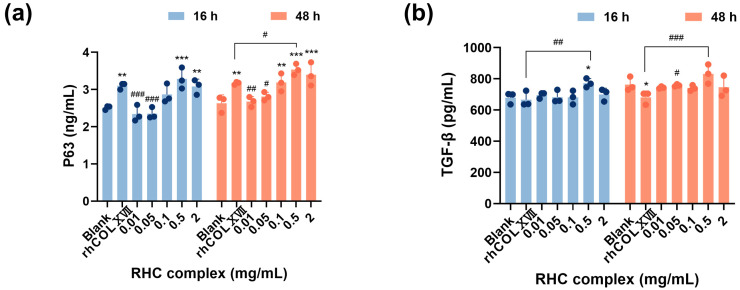
Expression of cell growth regulators in UVC-damaged HFF-1 cells. (**a**) P63 expression level in UVC-damaged HFF-1 cell cultures treated with different concentrations of the RHC complex. (**b**) TGF-β expression level in UVC-damaged HFF-1 cell cultures treated with different concentrations of the RHC complex. All groups were irradiated (UVC = 0.3 J/cm^2^), the rhCOLXVII group used culture media containing 0.5 mg/mL rhCOLXVII, and the blank group used culture medium containing 0.5 mg/mL RHC complex. Data represent the mean of *n* = 3 replicates ± SD. (* *p* < 0.05, ** *p* < 0.01 and *** *p* < 0.001 compared with blank group; # *p* < 0.05, ## *p* < 0.01, ### *p* < 0.01 compared with rhCOLXVII group).

To further explore the growth regulation mechanism of the RHC complex in UVC-damaged HFF-1 cells, we conducted an analysis of P63 and TGF-β levels in cell cultures using ELISA. The ELISA results (Figure 4a) demonstrated that the secretion of P63 by the damaged cells treated with the RHC complex generally increased over time. Importantly, the pro-secretory effects observed in the concentration groups of 0.5 and 2 mg/mL for P63 were significantly greater than those in the blank group. However, this effect did not reach statistical significance compared to the control group (0.5 mg/mL rhCOLXVII) at 16 h of treatment. Additionally, a positive correlation was noted between P63 content and sample concentration.

To explore whether the RHC complex can promote the TGF-β secretion from human fibroblasts damaged by UVC exposure, we measured TGF-β. Figure 4b shows that, compared to the blank group (0 mg/mL) and the control group (0.5 mg/mL rhCOLXVII), samples of 0.5 mg/mL had a boosting effect on TGF-B after 16 and 48 h of treatment. Overall, the secretion of TGF by UVC-injured HFF-1 cells increased over time.

### 3.4. The RHC Complex Reinforcing Effect of the BM in a Dorsal Rat Skin Model

To further investigate the efficacy of the RHC complex (rhCOLI:rhCOLIII:rhCOLXVII:Nicotinamide = 50:200:5:1) on the basement membrane (BM), the BM proteins and BM activity were analyzed after in vivo treatment.

**Figure 5:**
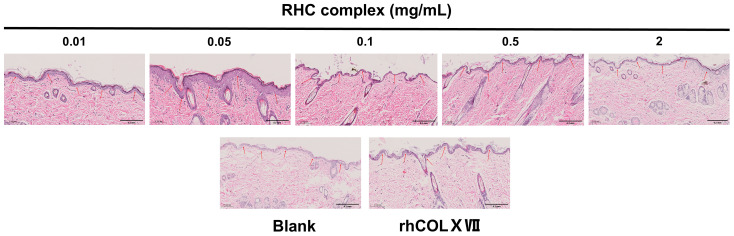
Comparison of different concentrations of the RHC complex with rhColXVII (0.5 mg/mL) and blank (0 mg/mL) groups on BM. Representative images of the H&E-stained transverse sections of skin tissues on day 7. (scale bar = 0.2 mm). The red arrows in the images are labeled as the BM located at the junction of the epidermis and connective tissue. Observation of the folds of the BM can reflect the bioactivity of the BM.

To assess the role of the RHC complex in maintaining the bioactivity of the BM, HE histochemical staining was conducted on isolated tissues. As shown in Figure 5, the thickness of the epidermal layer after treatment with different concentrations of the sample was heterogeneous and wavy distributed in all groups. In comparison to the blank group (0 mg/mL), the BM in the groups treated with various concentrations of the RHC complex exhibited more pronounced curvature and folding. Notably, when compared to the control group (0.5 mg/mL rhCOLXVII), the culture medium concentration groups showed even more significant curvature and folding, resembling the characteristics of control skin tissue. Reinforcing the BM can facilitate the growth and differentiation of epidermal cells, which may lead to the thickening of the epidermal layer [26], and the epidermal thickness of the group with a concentration of 0.05 mg/mL was significantly thicker. These results suggested that the RHC complex was beneficial for enhancing BM bioactivity, with the most pronounced effects observed at concentrations of 0.05, 0.01, and 0.5 mg/mL.

#### The RHC Complex Promoted the Expression of Laminin, Perlecan, and Integrin Proteins

**Figure 6:**
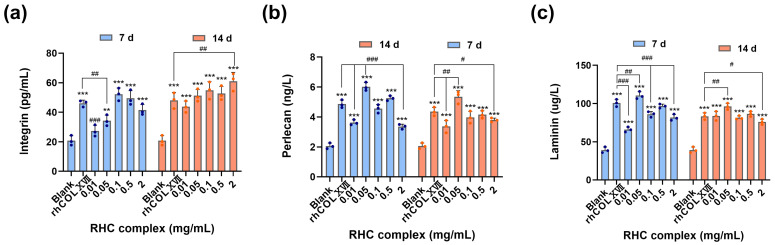
Expression of (**a**) integrin, (**b**) laminin, and (**c**) perlecan proteins in rats’ skin by ELISA with different concentrations of the RHC complex. The rhCOLXVII (0.5 mg/mL) group was used as a control group, and the blank group used 0 mg/mL of RHC complex. Data represent the mean of *n* = 3 replicates ± SD. (** *p* < 0.01, and *** *p* < 0.001 compared with blank group; # *p* < 0.05, ## *p* < 0.01, ### *p* < 0.01 compared with rhCOLXVII group).

**Figure 7:**
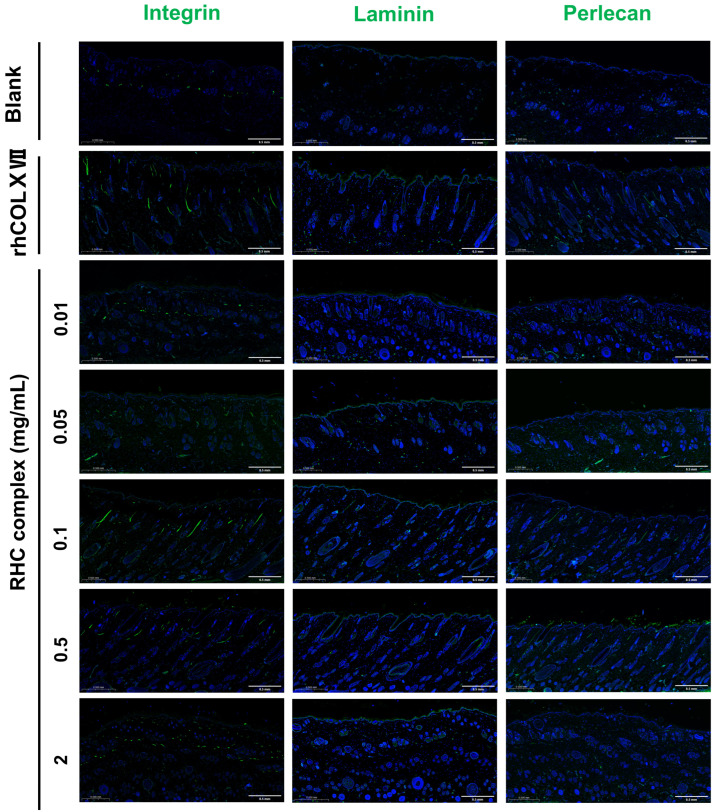
Images of IF labeling of isolated skin tissues with integrin (green), laminin (green), perlecan protein (green), and DAPI (blue), which are treated with different concentrations of the RHC complex for 7 days compared to rhCol XVII (0.5 mg/mL) and blank (0 mg/mL) groups. (Scale bar: 0.5 mm).

LN, integrin, and perlecan are essential for the maintenance of BM activity. The effect of the RHC complex on the expression of BM proteins was assessed by ELISA (Figure 6a–c). The results indicated that the different concentrations of the RHC complex enhanced the expression of all three proteins, with optimal expression observed across varying concentrations compared to the blank group. For a dosing cycle of 7 days, the RHC complex concentration of 0.05 mg/mL was particularly effective in promoting the expression of LN and perlecan. In contrast, a media concentration of 0.1–0.5 mg/mL was more effective for integrin expression. When the dosage period was 14 days, different concentrations of the RHC complex promoted the expression of three BM proteins, especially 0.05 mg/mL. In addition, the overall effect was more significant when the dosing period was 7 days. To determine the expression of LN, integrin, and perlecan proteins in the treated tissues nd evaluate the effect of the RHC complex on the maintenance of BM activity, isolated tissues were collected after the early stage of the drug treatment and subjected to IF staining. As shown in Figure 7, different concentrations of the RHC complex promoted the expression of LN, integrin, and perlecan proteins to a certain extent compared with the blank group (0 mg/mL), which was higher than the control group (0.5 mg/mL rhCOLXVII). The pro-secretory effects of recombinant the RHC complex at concentrations of 0.05, 0.01, and 0.5 mg/mL on BM proteins were particularly pronounced after a 7-day administration cycle, which aligned with the results obtained from HE histochemical staining.

## 4. Discussion

RHC research is currently a significant area of interest within the cosmetic industry, primarily due to its safety benefits and lower risk of pathogenicity when compared to collagen sourced from animals [22]. Consequently, we developed an RHC complex and examined its potential reinforcing effects on skin basement membranes.

In contrast to previous studies, the innovation of this research is the preparation of a mixture containing RHC types I, III, and XVII, along with niacinamide. Type I collagen is responsible for approximately 85% of the collagen stores of the body and is found only below the surface of the skin in the dermis [27]. Type III collagen is synthesized by cells as a pre-procollagen. Type XVII collagen is a transmembrane collagen protein at the dermal-epidermal junction [28]. Type III collagen often contributes to mixed fibrils with type I collagen and is also abundant in elastic tissues. The hybrid fibrils were reported to have a COLI: COLIII ratio of 2:1 to 3:1 in the skin, and COLI-COLIII hybrid fibrils are favorable over COLIII fibrils for fibroblast activation [29]. Niacinamide has whitening properties and can also reduce ECM changes by increasing collagen, elastin, and fibrillin [30]. Therefore, we prepared a mixture of RHC types I, III, and XVII with nicotinamide in the ratio of 50:200:5:1 for backup and demonstrated that the mixture was not cytotoxic to HFF-1 cells.

**Figure 8:**
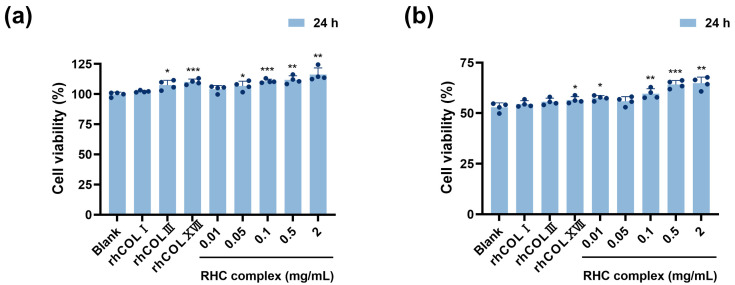
(**a**) Effect of RHC complex concentrations and single RHC on HFF-1 cell viability. (**b**) Effect of RHC complex concentrations and single RHC on HFF-1 UVC-damaged (UVC = 0.3 J/cm^2^) cells viability. The concentration of rhCOLI, rhCOLIII, and COLXVII is 0.5 mg/mL, and the concentration of the blank group is 0 mg/mL. Data represent mean of *n*= 4 replicates ± SD. (* *p* < 0.05, ** *p* < 0.01, and *** *p* < 0.001 compared with the blank group).

In its youthful state, the skin BM has a healthy and wavy shape, but it flattens with age. If the connections are completely flattened, signaling and nutrient exchange cease, resulting in a loss of skin elasticity and firmness [28]. The RHC complex exhibited the same activity as other collagens and their derivatives in reinforcing the skin BM, allowing the BM to exhibit a more pronounced curvature and folding and thickening of the epidermal layer [22,31]. The RHC complex and its single components had no cytotoxic effects, and higher concentrations of the complex promoted the proliferation of skin fibroblasts (Figure 8a). The core structural components of the BM are laminin, COLIV, nidogen, and heparin sulfate proteoglycans (HSPGs, including perlecan, agrin, etc.) [32]. Perlecan, a proteoglycan found in the ECM of the BM, assembles into the ECM along with nestin and acts as a bridging protein between COLVI and LN to form the BM [33,34,35]. Integrins are transmembrane heterodimeric proteins that help stabilize cell attachment to BM by binding to specific ligands on the BM, such as LN, FN, and collagen [36]. In this study, the ELISA and IF experiments confirmed that the RHC complex promoted the expression of LN, integrins, and perlecan in the BM of rat skin, reinforcing the BM structure, which precisely explained the results of HE tissue staining.

UV injury can lead to a decrease in the number of fibroblasts, and a reduction in their ability to synthesize collagen and elastin, further resulting in BM damage and degeneration of the epidermis and dermis [37]. The dense layer of the damaged skin BM is severely damaged, with multiple loose layers, a decrease in anchoring fiber-associated proteins, and a weakening of the adhesion between the epidermis and the dermis [38,39]. We constructed a simulated BM layer using HFF-1 cells in vitro and utilized UVC radiation to achieve the goal of damaging the BM layer. Various concentrations of the complex led to a reduction in the inhibition of damaged HFF-1 cell proliferation, with the high-concentration group exhibiting a more pronounced effect. Additionally, the inhibitory impact of the complex was notably greater than that of any individual component (Figure 8b). The marked decrease in the overall survival rate of cells after 48 h compared to 16 h may be attributed to the prolonged damage inflicted by ultraviolet radiation on the cells [40]. The findings can be confirmed through the CCK8 experiment, which demonstrated that the cell viability in the blank group declined by roughly 20% after 48 h in comparison to 16 h. Furthermore, we employed a serum-free culture medium to minimize any potential interference, as extended periods of cell fasting may also lead to cellular damage [41,42].

The growth of skin fibroblasts is multiply regulated by a variety of growth factors, including basic fibroblast growth factor (b-FGF), transforming growth factor-β (TGF-β), epidermal growth factor (EGF), etc. [43,44]. Exposure to a variety of external stimuli can lead to the apoptosis of skin cells, the most important of which is UV radiation [45]. TGF-β is a major regulator of ECM biosynthesis and can regulate collagen synthesis and degradation via the Smad pathway in skin fibroblasts [46,47]. The cell stemness factor P63 plays a crucial role in skin development and regulates cellular self-renewal mainly through the modulation of several components in the signaling pathway, including Hedgehog (Hh), Notch, TGF-β/Smad, and WNT/b-catenin [48,49]. Overall, the levels of TGF-β and P63 secreted by UVC-damaged cells after 16 and 48 h were positively correlated with the concentration of drug treatment. This precisely indicated that the treatment of the RHC complex promoted the secretion of TGF-β and P63 in skin fibroblasts, alleviated the overall shedding of damaged cells, and enhanced the damaged BM. Previous studies demonstrated that the lack of both P63 and TGF-β decreased cell survival and lowered collagen synthesis [50,51], which aligned with our findings.

Our results also indicated that high concentrations of the RHC complex can stimulate damaged cells to secrete COLI and COLIV, thereby alleviating cell shedding. Wang [52] demonstrated that the implantation of recombinant humanized type III collagen led to increased expression of both COLI and COLIII in UV-injured skin. FN and LN are important non-collagenous glycol-proteins in the ECM and BM. FN determines cell adhesion, spreading, migration, proliferation, and apoptosis and is a major protein for signaling in the BM and basal dermis [53,54]. LN interacts with other ECM proteins and binds to and activates cell surface proteins that transduce molecular signals, promoting BM assembly [54,55]. The research conducted by Lukasz et al. established a notable relationship between FN and COLIV/LN and between LN-5 and COLIV [31]. Our results found that high concentrations of the RHC complex promoted COLIV and LN secretion in injured HFF-1 cells, emphasizing the role of specific binding interactions between LN and collagen in facilitating the upregulation of collagen. Nevertheless, our data revealed that the RHC complex did not significantly increase FN expression in the damaged cells. The growth-enhancing effect of the RHC complex on UV-injured cells may be linked to other proteins that interact with FN, including heparan sulfate and integrins [54].

Our study prepared an RHC complex and explored its potential mechanism on BM by constructing a rat skin model and simulating the BM layer with UVC damage. However, this study did not address whether the complex exerts an inhibitory effect on interstitial collagenase, which is mainly secreted by fibroblasts and macrophages and plays a crucial role in the degradation of collagen types I, II, and III in the skin [56,57]. Furthermore, under our present experimental conditions, the direct penetration of UVC damage through the epidermal layer to the basement membrane at the carrier level is not feasible. Additionally, we did not perform UVC damage experiments at the animal level, which represents another limitation of our study.

## 5. Conclusions

In this study, we prepared a recombinant humanized collagen (RHC) complex composed of rhCOLI, rhCOLIII, and rhCOLXVII with niacinamide in the ratio of 50:200:5:1. The complex was found to be non-cytotoxic, as demonstrated by the CCK-8 assay. The RHC complex significantly reduced the proliferation inhibition of UVC-injured HFF-1 cells and increased the expression of COL I and COL IV. These effects may be attributed to the increased expression levels of LN, TGF-β, and P63. The most pronounced effects were observed at high concentrations (0.5 and 2 mg/mL) when samples were treated for 16 h. Additionally, the RHC complex significantly increased BM activity, with the reinforcing effect being more pronounced at 7 days of treatment in the medium concentration group (0.05, 0.01, and 0.5 mg/mL). This may be due to the upregulation of integrin, laminin, and perlecan expression. Overall, the findings indicated that the RHC complex promoted the secretion of fibroblast and BM proteins, facilitated the reinforcement of the skin BM, and may be a promising material for skin care products that reinforce and repair the skin BM.

## Figures and Tables

**Table 1:** The group of rat experiments.

Type of Group	RHC Dose	Number of Rats
Blank	0 mg/mL	6
Control	0.5 mg/mL rhCOLXVII	6
Experimental	0.01 mg/mL RHC complex	6
	0.05 mg/mL RHC complex	6
	0.1 mg/mL RHC complex	6
	0.5 mg/mL RHC complex	6
	2 mg/mL RHC complex	6

**Table 2:** The group of cell experiments.

Type of Group	RHC Dose	UVC Radiation Dose
Blank	0 mg/mL	0.3 J/cm^2^
Control	0.5 mg/mL rhCOLXVII	0.3 J/cm^2^
Experimental	0.01 mg/mL RHC complex	0.3 J/cm^2^
	0.05 mg/mL RHC complex	0.3 J/cm^2^
	0.1 mg/mL RHC complex	0.3 J/cm^2^
	0.5 mg/mL RHC complex	0.3 J/cm^2^
	2 mg/mL RHC complex	0.3 J/cm^2^

## Data Availability

The data supporting the findings of this study are available upon reasonable request from the corresponding authors.

## Supplementary Materials

The following supporting information can be downloaded at https://www.mdpi.com/article/10.3390/jfb16020047/s1, Supplementary Table S1: Recombinant Humanized Collagen Information.

## Abbreviations

The following abbreviations are used in this manuscript:

ECM	extracellular matrix
RHC	recombinant humanized collagen
BM	basement membrane
rhCOLI	recombinant humanized type I collagen
rhCOLIII	recombinant humanized type III collagen
rhCOLXVII	recombinant humanized type XVII collagen
COLI	type I collagen
COLIII	type III collagen
COLIV	type IV collagen
FN	fibronectin
LN	laminin
