# Supplementary material for: Recombinant Humanized Collagen Enhances Secreted Protein Levels of Fibroblasts and Facilitates Rats’ Skin Basement Membrane Reinforcement
Source: J Funct Biomater. 2025 Feb 1;16(2):47. doi: 10.3390/jfb16020047 (PMC11856143; doi:10.3390/jfb16020047)
Supplement: Supplementary file 1 [file jfb-16-00047-s001.zip › jfb-3409624-supplementary.pdf]

Supplementary Table S1. Recombinant Humanized Collagen Information

Type	Name	Molecular weight	Amino acid sequence
rhCOLI	ReCol1-TTC01-02SC	110~120 kDa	QLSYGYDEKSTGGISVPGPM<br>GPSGPRGLPGPPGAPGPQGF<br>QGPPGEPGEPGASGPMGPRG<br>PPGPPGKNGDDGEAGKPGRP<br>GERGPPGPQGARGLPGTAGL<br>PGMKGHRGFSGLDGAKGDAG<br>PAGPKGEPGSPGENGAPGQM<br>GPRGLPGERGRPGAPGPAGA<br>RGNDGATGAAGPPGPTGPAG<br>PPGFPGAVGAKGEAGPQGPR<br>GSEGPQGVRGEPGPPGPAGA<br>AGPAGNPGADGQPGAKGANG<br>APGIAGAPGFPGARGPSGPQ<br>GPGGPPGPKGNSGEPGAPGS<br>KGDTGAKGEPGPVGVQGPPG<br>PAGEEGKRGARGEPGPTGLP<br>GPPGERGGPGSRGFPGADGV<br>AGPKGPAGERGSPGPAGPKG<br>SPGEAGRPGEAGLPGAKGLT<br>GSPGSPGPDGKTGPPGPAGQ<br>DGRPGPPGPPGARGQAGVMG<br>FPGPKGAAGEPGKAGERGVP<br>GPPGAVGPAGKDGEAGAQGP<br>PGPAGPAGERGEQGPAGSPG<br>FQGLPGPAGPPGEAGKPGEQ<br>GVPGDLGAPGPSGARGERGF<br>PGERGVQGPPGPAGPRGANG<br>APGNDGAKGDAGAPGAPGSQ<br>GAPGLQGMPGERGAAGLPGP<br>KGDRGDAGPKGADGSPGKDG<br>VRGLTGPIGPPGPAGAPGDK<br>GESGPSGPAGPTGARGAPGD<br>RGEPGPPGPAGFAGPPGADG<br>QPGAKGEPGDAGAKGDAGPP<br>GPAGPAGPPGPIGNVGAPGA<br>KGARGSAGPPGATGFPGAAG<br>RVGPPGPSGNAGPPGPPGPA<br>GKEGGKGPRGETGPAGRPGE<br>VGPPGPPGPAGEKGSPGADG<br>PAGAPGTPGPQGIAGQRGVV<br>GLPGQRGERGFPGLPGPSGE

			PGKQGPSGASGERGPPGPM
			GPPGLAGPPGESGREGAPGA
			EGSPGRDGSPGAKGDRGETG
			PAGPPGAPGAPGAPGPVGPA
			GKSGDRGETGPAGPAGPVGP
			VGARGPAGPQGPRGDKGETG
			EQGDRGIKGHRGFSGLQGPP
			GPPGSPGEQGPSGASGPAGP
			RGPPGSAGAPGKDGLNGLPG
			PIGPPGPRGRTGDAGPVGPP
			GPPGPPGPPGPPSAGFDFSF
			LPQPPQEKAHDGGRYYRA
rhCOLIII	ReCol3-TTA01-	10~55 kDa	AGNTGAPGSPGVSGPKGDAG
	02PC		QPGEKGSPGAQGPPGAPGPL
			GIAGITGARGLAGPPGMPGP
			RGSPGPQGVKGESGKPGANG
			LSGERGPPGPQGLPGLAGTA
			GEPGRDGNPGSDGLPGRDGS
			PGGKGDRGENGSPGAPGAPG
			HPGPPGPVGPAGKSGDRGES
			GPAGPAGAPGPAGSRGAPGP
			QGPRGDKGETGERGAAGIKG
			HRGFPGNPGAPGSPGPAGQQ
			GAIGSPGPAEFTAGNTGAPG
			SPGVSGPKGDAGQPGEKGSP
			GAQGPPGAPGPLGIAGITGA
			RGLAGPPGMPGPRGSPGPQG
			VKGESGKPGANGLSGERGPP
			GPQGLPGLAGTAGEPGRDGN
			PGSDGLPGRDGSPGGKGDRG
			ENGSPGAPGAPGHPGPPGPV
			GPAGKSGDRGESGPAGPAGA
			PGPAGSRGAPGPQGPRGDKG
			ETGERGAAGIKGHRGFPGNP
			GAPGSPGPAGQQGAIGSPGP
			ADHHHHHHT LARF
rhCOL XVII	TTB01-02SC	10~23.8 kDa	YVWSHPQFEKGSPGPKGDMG
			SPGPKGDRGFPGTPGIPGPL
			GHPGPQGPKGQKGSVGDPG
			EGPGEKGERGAAGEPGPHGP
			PGVPGSVGPKCSSGSPGPQG
			PPGPVGLQGLRGEVGLPGVK
			GDKGPMGPPGPKGDQGEKGP
			PGPPGPPGPKGDQGPPGPRG

	HQGEQGLPGF SGPPGPPGPQ<br>GPKGDKGDPGVPGALGIPGP<br>PGQKGEMGTPGPKGDRGPAG<br>PPGHPGPPGPRGHKGEKGDK<br>GDQHHHHHH
